# Case Study: Pseudobulbar Affect During Recovery From Locked in Syndrome

**DOI:** 10.1192/bjo.2024.652

**Published:** 2024-08-01

**Authors:** Sunil J. Ankolekar, Sheryl Parke

**Affiliations:** 1 Norfolk Community Health and Care NHS Trust, Norwich, United Kingdom; 2 University of East Anglia, Norwich, United Kingdom

## Abstract

**Aims:**

We would like to report a case of pseudo bulbar affect during recovery from locked in syndrome due to brainstem stroke.

**Methods:**

We present a lady in her early 60s who developed pseudobulbar affect during recovery from locked in syndrome. MRI brain confirmed brain stem infarct. No personal or family history of mental illnesses was noted. Neurological examination on our rehabilitation unit confirmed dense weakness in all four limbs. She would cry even when family gave her good news or made jokes with her. This appeared to be the only method of expressing her emotions she had; however, it was unclear if this was aligned to her internal emotional experience.

**Results:**

Through clinical observation and using the Testing Emotionalism After Recent Stroke-Questionnaire (TEARS-Q) measure of emotionalism we identified that pseudobulbar affect was present, and intervention should be considered. The patient also stated that her crying was not always aligned with her emotional experience, but laughter was. The Clinical Outcome Routine Evaluation (CORE-10) was also used to screen out other potential psychological difficulties including depression. The assessment indicated she was experiencing low levels of psychological distress.

We initiated fluoxetine and clonazepam was given to help with spasticity and sleep. Our non-pharmacological measures included sitting with the emotional expression and not asking her to stop, encouraging her to take deep breaths and modelling this and when she presented as calmer supporting her to identify if her emotional expression was in line with her internal emotional experience and using different communication strategies to explore this and support her to have her needs met. If the crying persisted mid communication, staff supporting her would reorientate her to what she had been attempting to communicate and encourage her to continue, which she would be able to do. All staff were asked to do this during their interactions with the patient to support identification of emotional alignment. Significant reduction in emotional misalignment was noted following the implementation and increased use of external communication aids. Within a few months her distressing crying episodes reduced and neurologically she improved.

**Conclusion:**

Pseudo bulbar affect is a distressing condition that can occur during recovery from locked in syndrome. Diagnosis can be confirmed by ruling out other common conditions like anxiety or depression. Treatment includes both non-pharmacological and pharmacological measures best provided by a specialist multidisciplinary team.

